# Supplementary material for: WordCommentsAnalyzer: A windows software tool for qualitative research
Source: F1000Res. 2018 Sep 4;7:536. Originally published 2018 May 3. [Version 2] doi: 10.12688/f1000research.14819.2 (PMC6137411; doi:10.12688/f1000research.14819.2)
Supplement: Supplementary file 3 [file f1000research-7-17614-s0002.tgz › b2a79496-4ada-47c4-9682-a99ce867836d.zip!/Eight Iranian Health Journals/The Journal of Toloo-e-Behdasht_32.docx]

© The Journal of Toloo-e-Behdasht. This work is licensed under CC BY 4.0 (https://creativecommons.org/licenses/by/4.0/).

Volume & Issue: دوره 11 - شماره 4

Title: پیش بینی ریسک ابتلا به عارضه ریوی در کارگران مواجهه با غبار سیلیس کارگاه های سنگ کوبی با استفاده از مدل رگرسیونی کاکس

http://tbj.ssu.ac.ir/article-1-351-fa.html

سابقه و اهداف : شرایط مواجهه شغلی کارگران از جمله تراکم غبار ، مدت زمان مواجهه، نحوه استفاده از وسایل حفاظت تنفسی و استعمال سیگار در ابتلاء کارگران به عارضه ریوی ناشی از مواجهه با غبار سیلیس تاثیر گذار است، هدف از مطالعه حاضر تعیین ریسک مخاطره ابتلاء به عوارض ریوی در کارگران سنگ کوبی با استفاده از تکنیک رگرسیون کاکس می باشد.   روش بررسی : در این مطالعه 117 نفر از کارگران شاغل در کارگاه سنگ کوبی استان همدان به روش تصادفی ساده انتخاب گردیدند. اطلاعات مواجهه شغلی کارگران با استفاده از پرسشنامه جمع آوری گردید و نتایج آزمون های عملکرد ریوی که با استفاده از دستگاه تنفس سنج مدل Spirotrav IV شرکت Vitalograph صورت گرفته بود،استخراج گردید. اطلاعات با استفاده از نرم افزار16 SPSS و مدل رگرسیونی کاکس تحلیل گردید.   یافته ها: براساس نتایج مدل متغیرهای سن، استعمال سیگار و استفاده از وسایل حفاظت تنفسی به عنوان متغیرهای تاثیرگذار بر ابتلاء به عارضه ریوی تعیین گردید. ریسک مخاطره ابتلاء در کارگران سیگاری 44/8 برابر کارگران غیرسیگاری بود. همچنین ریسک مخاطره ابتلاء در کارگرانی که از وسایل حفاظت فردی استفاده نمی کردند 3/3 برابر کارگران استفاده کننده از این وسایل تعیین گردید.   نتیجه گیری: استفاده از مدل کاکس به خوبی توانست شرایط مواجهه شغلی موثر بر ابتلاء کارگران به عارضه ریوی را تبیین نماید. بر این اساس پیشنهاد گردید با ایجاد بانک های اطلاعات پزشکی و با استفاده از مدل مذکور، ریسک مخاطره ابتلاء به بیماری های ناشی از کار که عمدتا غیر قابل درمان و البته قابل پیشگیری هستند، تعیین گردد تا براساس مقادیر ریسک نسبی متغیر های موثر بر ایجاد بیماری ها،مداخلات بهداشتی مورد نیاز پیش بینی گردد.    رگرسیون کاکس، عارضه ریوی، غبار سیلیس

Title: بررسی تاثیر توزیع سبد غذایی کمیته امداد امام خمینی(ره) بر وضعیت رشد کودکان 6-1 ساله

http://tbj.ssu.ac.ir/article-1-352-fa.html

سابقه و اهداف : سوء تغذیه کودکان از مشکلات شایع سلامتی است که ریشه در عوامل متعددی دارد. یکی از این عوامل فقر غذایی است. کودکی که از غذای کافی جهت تأمین نیازهای غذایی خود بهره مند نگردد، در نهایت مبتلا به سوء تغذیه و اختلال رشد خواهد شد. این مطالعه با هدف تعیین اثر توزیع سبد غذایی ماهیانه بین خانواده های دارای کودک مبتلا به سوء تغذیه بر بهبود این مشکل در کودکان آنها انجام شده است.   روش بررسی: این مطالعه به عنوان یک کارازمایی میدانی با مشارکت 300 نفر کودک 6-1 ساله مبتلا به اختلال رشد در شهرستان های خاتم و تفت از شهرهای استان یزد انجام شد. 150 کودک در منطقه روستایی شهرستان خاتم تحت آموزش تغذیه و سبد غذایی ماهیانه قرار گرفتند، در حالیکه 150 کودک در منطقه روستایی شهرستان تفت صرفاً تحت آموزش تغذیه قرار گرفتند. داده های تن سنجی شامل قد و وزن در آغاز، شش ماه و یکسال بعد از مداخله ثبت و شاخص های مختلف مثل امتیاز Z وزن و قد برای سن محاسبه و با استفاده از بسته نرم افزاری SPSS مورد تجزیه و تحلیل قرار گرفتند.   یافته ها: 161 نفر ( 6/53%) از افراد مورد مطالعه دختر و 139 نفر (4/46%) پسر بودند. میانگین امتیاز z وزن برای سن، قد برای سن و وزن برای قد، همچنین میانگین قد و وزن افراد مورد مطالعه در بدو مداخله، شش ماه و یکسال بعد از مداخله بین دو گروه مورد مطالعه تفاوت معنی داری نشان نداد.   نتیجه گیری: این مطالعه نشان داد حمایت های مستقیم مواد غذایی به حانواده هایی که کودک ابتلا به سوء تغیه دارند چندان ناثیری در بهبود این مشکل در کودک آنها نخواهد داشت و بهتر است شیوه های دیگری برای رفع این مشکل مورد توحه قرار گیرد.

Title: بهینه سازی متغیرهای تکنولوژی تغلیظ جهت تصفیه و کاهش بار آلی پساب صنعت الکل سازی

http://tbj.ssu.ac.ir/article-1-353-fa.html

سابقه و اهداف: پساب واحدهای تولید الکل به دلیل داشتن بار آلی زیاد و بالا بودن شاخص های COD و BOD قابل دفع مستقیم به محیط زیست نیست و تصفیه آن هزینه های هنگفتی را به واحد های تولیدی تحمیل می کند. هدف از این تحقیق کاربردی-تحقیقی کاهش بار آلی فاضلاب کارخانه الکل سازی با تکنولوژی تغلیظ است.   روش بررسی: در این پژوهش از تکنولوژی تغلیظ ویناس برای کاهش بار آلی و حذف رنگ و بوی فاضلاب استفاده گردید. در این فرایند سه واحد تبخیرکننده و سه واحد جداکننده بخار درنظر گرفته شد و شرایط به گونه ای تنظیم گردید که فاضلاب خام در بدو ورود به سیستم از مرحله ای با فشار بیشتر به مرحله بعدی با فشار کمتر منتقل می شود. از پساب خروجی هر مرحله نمونه برداری شد و میزان COD و BOD آن اندازه گیری شد.   یافته ها: نتایج مربوط به نمونه برداری پساب از خروجی فرایند در مراحل اول تا سوم و انجام آزمایش COD نشان می دهد که میزان حذف آن به ترتیب 98/68 ، 69/80 و 97/99 درصد بوده است. این نتایج برای آزمایش BOD به ترتیب 75/68، 68/85 و 97/99 درصد بوده است. باعبور فاضلاب از مراحل اول تا سوم، به تدریج رنگ و بوی فاضلاب نیز به تدریج حذف گردید.   نتیجه گیری : باتوجه به کارایی بسیار خوب تکنولوژی تغلیظ در کاهش بار آلی و حذف رنگ و بوی فاضلاب کارخانه الکل سازی و ارجحیت آن از نظر تولید خوراک دام و سوخت به عنوان محصول جانبی، استفاده از این روش برای تصفیه فاضلاب صنایع الکل سازی و صنایع مشابه پیشنهاد می شود.    پساب الکل سازی، بار آلی، تبخیر کننده ، جداکننده فاز بخار، تکنولوژی تغلیظ

Title: بررسی میزان جیوه موجود در کنسرو تن ماهی های عرضه شده در فروشگاههای مواد غذایی کرمان

http://tbj.ssu.ac.ir/article-1-354-fa.html

سابقه و اهداف: به علت دفع پساب های صنعتی به محیط های دریایی، مقدار زمینه فلزات سنگین و تجمع زیستی آنها در بدن موجودات دریایی از جمله ماهی ها رو به افزایش است. از این رو بررسی میزان فلزات سنگین در بدن ماهی ها که مورد استفاده غذایی انسان قرار می گیرند امری ضروری می نماید.   روش بررسی: در این مطالعه به منظور برآورد میزان جیوه در تن های ماهی عرضه شده در فروشگاههای مواد غذایی کرمان، تعداد 70 نمونه تن ماهی تهیه و مقدار جیوه آنها با استفاده از دستگاه ICP-OES تعیین شد. مقدار جیوه در نمونه ها بر اساس µg g-1 وزن مرطوب گزارش شد.   یافته ها: مقدار جیوه در 70 نمونه تن ماهی مورد بررسی از مقادیر 07/0 ± 1/0 تا µg g-1 2/0 ± 54/0 متغیر و میزان متوسط آن برابر با µg g-1 29/0 بود. نتایج حاصل از این مطالعه نشان داد که 80 درصد نمونه های تن ماهی (8 نوع) از ناحیه خلیج فارس ایران دارای میانگین مقادیر جیوه کمتر و 20 درصد (2 نوع) دارای میانگین مقادیر بالاتر از رهنمود های پیشنهادی WHO و FAO می باشند.   نتیجه گیری: نتایج حاصل از این مطالعه نشان داد که بیشتر تن ماهی های عرضه شده در فروشگاههای کرمان دارای میانگین مقادیر کمتر از رهنمودهای سازمان جهانی بهداشت و سازمان غذا و داروی آمریکا یعنی میزان µg g-1 5/0 می باشند ولی در 2 نمونه میانگین مقادیر بالاتر از رهنمودهای مذکور بود. در این زمینه بایستی تمهیدات لازم برای پایش مقادیر جیوه در تن ماهی های تولیدی در کشور و ملزوم نمودن کارخانجات تولیدی به پایش مقادیر جیوه و همچنین تدوین استانداردهای لازم برای پایش این فلز سنگین خطرناک در مواد غذایی اندیشیده شود.

Title: بررسی اثر استابیلایزر و طعم دهنده ها بر روی ویژگی های حسی ماست سویا

http://tbj.ssu.ac.ir/article-1-355-fa.html

سابقه و اهداف : تامین غذای مورد نیاز جهان،معضلی است که امروزه بشریت با همه توان خود در مقابل آن عاجز مانده است. امروزه در اغلب کشورها،فرآورده های غذایی سویا جایگاه خاصی را در رژیمهای غذایی به خود اختصاص داده اند. قیمت نسبتاً پایین،عدم وجود کلسترول و لاکتوز، وجود چربی های غیر اشباع و ضروری و به علاوه،کاهش چربی و کلسترول خون و نیز ممانعت از بروز تصلب شرائین از جمله فواید و خصوصیات بارز فرآورده های غذایی سویا می باشد . هدف این پروژه ارزیابی خصوصیات حسی ماست سویا بویژه طعم و بافت و بهبود میزان پذیرش آن می باشد که این هدف با افزودن طعم دهنده های موز،پرتقال،توت فرنگی و نشاسته اصلاح شده صورت گرفته است.   روش بررسی :این مطالعه با توجه به ارزیابی حسی توسط120 نفر از دانشجویان انجام گرفت. شیوه تحقیق در جمع آوری اطلاعات پرسشنامه بود. ازنشاسته ی اصلاح شده در غلظت های متفاوت(5/0- 75/0- 1 و25/1 درصد) و طعم دهنده ها در غلظت 5% استفاده وسپس بهترین بافت و طعم مورد پذیرش و میزان سینرزیس نمونه های حاوی 25/1 درصد نشاسته اصلاح شده در روزهای مختلف باتوجه به تست های آماری Kruskal-Wallis و ANOVA مورد ارزیابی قرار گرفتند.   یافته ها : ارزیابی های حسی طعم وبافت در روز 7 نگهداری انجام گرفت و نتایج نشان داد که بیشترین امتیاز پذیرش بافت به ترتیب مربوط به غلظت های 1- 75/0- 5/0 و 25/1 درصد و بیشترین امتیاز پذیرش طعم به ترتیب مربوط به طعم دهنده های پرتقال، موز و توت فرنگی می باشد.   نتیجه گیری : استفاده از طعم دهنده در ماست سویا باعث می شود که ضمن پوشاندن طعم نامطلوب، تنوعی فراوان در محصولات حاصله ایجاد شود. همچنین با توجه به اینکه ظرفیت اتصال آب پروتئین های گیاهی و حیوانی با هم اختلاف دارد،لذا استفاده از یک پایدارکننده می تواند در بهبود خصوصیات ظاهری بویژه بافت ماست سویا موثر باشد.   ماست سویا،طعم دهنده،استابیلایزر

Title: بررسی میزان شوینده ها در آبهای آشامیدنی شهر شیراز

http://tbj.ssu.ac.ir/article-1-356-fa.html

سابقه و اهداف: استفاده وسیع از شوینده ها از یک سو و عدم امکان دفع آن از سوی دیگر، امکان ورود این مواد را به آبهای زیرزمینی افزایش می دهد. این مواد علاوه بر ایجاد مسمومیت در حیوانات باعث ایجاد بیماریهای آلرژیک در انسان نیز می‌شود. هدف این مطالعه تعیین روند الودگی ابهای اشامیدنی شهر شیراز به شوینده ها می باشد.   روش بررسی: در این مطالعه توصیفی، تمام منابع آب آشامیدنی شهر شیراز (38 منبع) طی دو سال 87 و90مورد آزمایش قرار گرفت. اطلاعات مربوط به نمونه آب در یک چک لیست ثبت و نمونه ها پس از انتقال به آزمایشگاه توسط دستگاه پلاریمترِ ولتا آزمایش شد.   یافته ها: مقادیر تمام شوینده‌ها در حد استاندارد و رو به افزایش بود. آزمونهای آماری نشان داد که میزان شوینده‌ها در چاه‌های آبرفتی به طور معنی داری بیش از چاه‌های آهکی و شبکه آب آشامیدنی بود. بین میزان شوینده ها و سایر عوامل فیزیکی و شیمیایی ارتباط معنی داری مشاهده نشد.   نتیجه گیری: اگر چه میزان شوینده و فسفات در حال افزایش بود، اما در هیچ‌کدام از منابع آب شهر شیراز، میزان فسفات و شوینده بیش از حد استاندارد نبود، ولی به دلیل میزان بالای نیتریت و نیترات و روند رو به افزایش میزان شوینده و فسفات ، حفاظت و رعایت حریم بهداشتی چاه‌ها و تصفیه فاضلابها ضروری است.

Title: ارزیابی میزان آگاهی، نگرش و عملکرد دانش آموزان دبیرستانی شهر یزد در مورد نقش ویتامینD در سلامت

http://tbj.ssu.ac.ir/article-1-357-fa.html

سابقه و اهداف :ویتامین ها مواد آلی هستند که به مقدار کم مورد نیاز بدن بوده و کمبود هر یک از آن ها می تواند کل بدن را به مخاطره اندازد . شیوع کمبود ویتامین D در خاور میانه هشدار دهنده و در سراسر جهان اپیدمی شده است و علت اصلی این بیماری همه گیر ، فقدان قرارگیری در معرض نور آفتاب است.   این مطالعه به منظور تعیین میزان آگاهی، نگرش و عملکرد دانش آموزان دبیرستانی شهرستان یزد در مورد نقش ویتامین D در سلامت انجام شد .   روش بررسی :در این مطالعه توصیفی – مقطعی ، 312 نفر از دانش آموزان (153 پسر ، 159 دختر) از 8 دبیرستان شهر یزد ، که به روش طبقه ای و تخصیص مناسب و بصورت تصادفی در داخل هر طبقه انتخاب شدند، شرکت کردند. با استفاده از یک پرسشنامه ی محقق ساخته ی خودایفا و بی نام ، سطح آگاهی، نگرش و عملکرد دانش آموزان مورد سنجش قرار گرفت. داده ها با استفاده از نرم افزار spss با کمک آزمون های Paired t-test ، ANOVA و Chi2 تجزیه و تحلیل گردید .   یافته ها: از مجموع شرکت کنندگان 49 درصد پسر و 51 درصد دختر بودند.2/2 درصد از دانش آموزان دارای آگاهی خوب و 4/39 آگاهی متوسط داشتند.5/21 درصد آنها نگرش و 8/28 درصد عملکرد خوبی داشتند.میانگین نمره آگاهی، نگرش و عملکرد دانش آموزان در مورد نقش ویتامین D در سلامت به ترتیب 73/4±11/15از31 امتیاز و32/3± 94/18از 28 امتیاز و 71/2±69/13از20 امتیاز بود . بین وضعیت آگاهی و نگرش در دو جنس تفاوت معنی داری وجود نداشت ولی وضعیت عملکرد پسران بهتر از دختران بود و تفاوت معنی داری بین عملکرد پسران و دختران مشاهده گردید .   نتیجه گیری: کاستی های موجود در آگاهی و بدنبال آن نگرش و عملکرد می تواند سطح سلامت افراد را به خطر اندازد. بنابراین توصیه می شود برنامه های آموزشی مختلف در این رابطه برگزار گردد.

Title: بررسی امکان تجزیه بیولوژیکی پرکلرواتیلن (PCE) در غلظت بالا بوسیله راکتور بی هوازی با بستر سیال (AMBR)

http://tbj.ssu.ac.ir/article-1-358-fa.html

سابقه و اهداف: پرکلرواتیلن (PCE) از دسته هیدروکربن های آلیفاتیک کلردار است که کاربرد گسترده ای در صنایع دارد. پساب های صنعتی حاوی PCE به دلیل خطرات احتمالی ناشی از سرطانزایی آن باید تصفیه شوند. هدف از این مطالعه تعیین قابلیت تجزیه بیولوژیکی پرکلرواتیلن در غلظت بالا توسط یک راکتور بی هوازی با بستر سیال (AMBR) می باشد که تاکنون برای تجزیه بیولوژیکی PCE مورد استفاده قرار نگرفته است. روش بررسی: در این مطالعه یک راکتور بیهوازی با بستر سیال (AMBR) در مقیاس آزمایشگاهی به حجم کل 10 لیتر با 4 محفظه برای تجزیه بیولوژیکی پرکلرواتیلن در سوبستره سنتتیک مورد استفاده قرار گرفت. راه اندازی راکتور به کمک لجن هاضم بیهوازی انجام شد. پس از راه اندازی، کارایی راکتور در تجزیه PCE در غلظت mg/L 100 مورد بررسی قرار گرفت. زمان ماند هیدرولیکی (HRT) برابر 32 ساعت ثابت نگه داشته شد. یافته ها: بهترین بازده حذف COD به میزان 98% در بارگذاری آلی COD معادل g COD/L.d 1/3 بدست آمد. میانگین بازده حذف COD در کل دوره فعالیت راکتور به ترتیب برابر 4/91% بود. برای حذف PCE نیز بازده به میزان 5/99% حاصل شد. نتیجه گیری: نتایج نشان داد که راکتور بیهوازی با بستر سیال (AMBR)، در صورت انجام مطالعات تکمیلی در مقیاس کامل و استفاده از پساب صنعتی واقعی آلوده به پرکلرواتیلن یک روش ساده، کارامد و قابل اطمینان برای تصفیه پسابهای صنعتی آلوده به این ترکیب حتی در غلظت های بالا می باشد.

Title: میزان تاثیر برنامه آموزشی تغذیه و قلب سالم به رابطین مراکز بهداشتی ودرمانی شهرستان یزد بر وضعیت آگاهی، نگرش و عملکرد مادران

http://tbj.ssu.ac.ir/article-1-359-fa.html

سابقه و اهداف: بیماری های قلبی و عروقی یکی از علل عمده مرگ و میر در سطح جهان بوده و به عنوان اولین عامل مرگ، یک سوم کل مرگ و میرها درجهان را به خود اختصاص می‌دهد. این بیماریها علاوه بر مرگ و میر بالا، عوارض قابل توجهی را نیز به جا می‌گذارند جهت پیشگیری از بیماریهای قلبی و عروقی نیاز به اجرای برنامه های آموزش می‌باشد یکی از عاملهای بالقوه جهت بالابردن آگاهی، نگرش و عملکرد مادران، رابطان بهداشتی می‌باشند. هدف از این مطالعه تعیین تاثیر آموزش رابطین زن برآگاهی و نگرش و عملکرد مادران می‌باشد. روش بررسی: این مطالعه کار آزمایی بالینی از نوع قبل و بعد میباشد و بر روی 310 نفر از مادران که توسط رابطین بهداشتی آموزش دیده اند انجام شده است. مادران و رابطین بصورت تصادفی انتخاب شدند. 6 ماه پس از اجرای برنامه آموزشی رابطین،تاثیر آموزش بر روی مادران سنجیده شد. جهت تجزیه و تحلیل اطلاعات از نرم افزار،16 SPSSاستفاده شد. از آمار های توصیفی جهت خلاصه سازی اطلاعات و آزمون تی زوجی و همبستگی پیرسون جهت تجزیه وتحلیل داده ها استفاده گردید. یافته‌ها: نتایج پژوهش نشان می‌دهد که میانگین نمره آگاهی مادران قبل از اجرای برنامه آموزشی از 38 نمره8/5 ±4/26 (4/69%) بود که با اجرای برنامه آموزشی به3/4± 2/31 (1/82%)رسید (000/0 =P). میانگین نمره نگرش مادران از30 نمره قبل از آموزش 3/4± 2/24 ( 6/80%) بود که بعد ازآموزش توسط رابطین بهداشتی به 8/4±6/27 (92%) رسید که رابطه معنی داری را نشان می دهد (000/0 =P)میانگین نمره عملکرد مادران مورد مطالعه قبل از آموزش از 40 نمره 9/2± 7/14 (7/37%) بود که با اجرای برنامه آموزشی به 8/2± 2/17 (1/44%) رسیده است و نتیجه آزمون T زوج با 000/0P = رابطه معنی داری را نشان می‌دهد. نتیجه گیری: در این پژوهش تاثیر آموزش رابطین بر مادران به ترتیب 7/12% افزایش درآگاهی، 4/11% افزایش در نگرش و 4/6% افزایش در عملکرد مادران مشاهده شده که بنظر می‌رسد جهت تغییر بیشتر در زمینه افزایش عملکرد مطالعات بیشتری مورد نیاز است. این مقاله حاصل از پایان نامه دانشجوی کارشناسی ارشد آموزش بهداشت دانشگاه علوم پزشکی شهید صدوقی می باشد.

Title: بررسی میزان رضایتمندی بیماران مراجعه کننده به بخش های بستری بیمارستان سوانح سوختگی شهیدصدوقی یزد در نه ماهه اول سال1390

http://tbj.ssu.ac.ir/article-1-361-fa.html

سابقه و اهداف: رضایتمندی بیمار مفهوم مهمی است که بعنوان یکی از حیطه های مهم حاکمیت خدمات بالینی مورد توجه قرار گرفته است. نظریات بیماران در حیطه کیفیت خدمات، آئینه ای صاف از نقاط قوت و ضعف ماست. به کار بستن راهکار های مناسب می تواند باعث ارتقاء کیفی خدمات و جلوگیری از اتلاف هزینه نیز بشود.   روش بررسی: این پژوهش یک مطالعه توصیفی- تحلیلی و مقطعی بود که جامعه مورد پژوهش بیماران پذیرش شده در بخش های بستری بیمارستان سوانح سوختگی در نه ماهه ی اول سال 1390 بودند. تعداد افراد نمونه 542 بیمار بود که با روش طبقه بندی با تخصیص مناسب از جامعه مورد نظر انتخاب شدند. ابزار گرد آوری اطلاعات پرسشنامه ا ی شامل 24 سؤال بود که با استفاده از مقیاس درجه بندی لیکرت مورد سنجش قرار گرفت. داده ها با استفاده از نرم افزارهای 18 Spss و16 Amos و روش های آماری توصیفی و استنباطی تجزیه و تحلیل شد.   یافته ها: به طور کلی 2/81% از مراجعین از خدمات ارائه شده رضایتمندی خوب،6/18% متوسط و 2/0 % رضایتمندی متوسط داشتند. بیشترین میزان رضایتمندی مربوط به خدمات پزشکی(3/93%) و کمترین رضایتمندی مربوط به امکانات رفاهی(80%) بود. طبق نتایج بدست آمده از آزمون تحلیل مسیر، اتاق عمل و امکانات و خدمات بخش بیشترین تأثیر را در میزان رضایت بیماران داشتند و همچنین بین متغیر های جنس، سطح تحصیلات و مدت اقامت با رضایت کلی بیمار ارتباط معنی داری وجود داشت.   نتیجه گیری: با توجه به ارتباط معنی دار میان شاخص های محیط و خدمات بیمارستان با رضایت بیماران می توان با برنامه ریزی در جهت بهبود وضعیت بیمارستان به افزایش رضایت بیماران اقدام نمود.

Title: بررسی فراوانی کاندیدیازیس دهانی درافراداستفاده کننده ازدست دندان درمراجعین به دانشکده دندانپزشکی شهریزد

http://tbj.ssu.ac.ir/article-1-363-fa.html

سابقه و اهداف: دنچر استوماتیت، از اشکال رایج کاندیدیازیس دهانی است که به صورت التهاب منتشردر نواحی که تحت پوشش دنچرماگزیلاری می باشد، دیده می شود. این عارضه درحداقل 70% افراد دارای دنچربانشانه های بالینی رشدقارچ نمایان می گرددو محتمل ترین علت آن کلونیزاسیون قارچ کاندیدا درمخاط دهان است. قارچ کاندیدا دردهان بویژه گونه کاندیدا آلبیکنس به عنوان یک عامل عفونت اندوژن، بدنبال ترومای مزمن ناشی از دنچر به مخاط صدمه دیده حمله کرده که ممکن است در افراد ایمنوساپرس منجر به عفونتهای فرصت طلب سیستمیک شود. هدف از انجام مطالعه حاضر بررسی میزان فراوانی کاندیدیازیس دهانی در افراد دارای دنچرکامل مراجعه کننده به دانشکده دندانپزشکی شهر یزد بوده است.   روش بررسی: این مطالعه توصیفی مقطعی پس از تکمیل پرسشنامه طراحی شده برروی 109 بیمار مراجعه کننده به دانشکده دندانپزشکی یزدکه دارای دنچر کامل بوده وبطورمرتب ازآن استفاده می نمودند، انجام شد. دهان این افراد ابتدا توسط یک متخصص بیماریهای دهان معاینه و سپس از نواحی مختلف دهان نمونه گیری انجام، و نمونه ها پس از تهیه لام مستقیم بلافاصله برروی محیط کشت سابورو دکستروز آگار کشت داده و در نهایت تعداد کلنی مخمرهای جدا شده از هر نمونه شمارش شدند.   یافته ها: تفاوت آماری معنی داری بین میزان کلونیزاسیون کاندیدا بر روی دنچر در دوجنس مرد و زن مشاهده نشد (32/0 P= ). شمارش تعداد کلنیهای کاندیدا جدا شده از نواحی معین دهان (کام ، گونه ، زبان) و نیزدرسطح دنچر درافراد موردمطالعه ، برحسب وجود و عدم وجود ضایعات دهانی انجام گردید. کلنی کانت کاندیدا درسطح مخاطی دنچر در افراد واجد ضایعه دهانی بطورمعنی داری بیشترازگروه فاقد ضایعه دهانی ( 02/0 P= ) بود و زبان واجد بیشترین میزان کلونیزاسیون کاندیدایی بودند. در این مطالعه، در سطح زبان و سطح ماگزیلاری دنچر بیشترین میزان جداسازی کاندیدا مشاهده شد.   بحث ونتیجه گیری: نتایج تحقیق حاضر نقش قوی کاندیدا دربروزضایعات دهانی بویژه استوماتیت ناشی از دنچرراتائیدمی نماید. رعایت بهداشت دهان و دنچر و در صورت لزوم ضد عفونی کردن هرروزه آن در افراد دارای دنچر می تواند در کاهش کلونیزاسیون کاندیدا و در نتیبجه کاهش کاندیدیازیس دهانی در آنها موثر باشد.

Title: بررسی شیوع و علل گرایش به مصرف سیگار و قلیان در دانشجویان دانشگاه علوم پزشکی تهران در سال تحصیلی 90-1389

http://tbj.ssu.ac.ir/article-1-362-fa.html

سابقه و اهداف: مصرف دخانیات به عنوان یک مشکل بهداشت عمومی درسطح جهان مطرح می باشد، همچنین میتواند موجب کاهش امید به زندگی ، افزایش هزینه درمانی، کاهش خلاقیت درابعاد زندگی فردی شود. این مطالعه با هدف بررسی مصرف سیگار و قلیان و علل گرایش به مصرف این مواد در دانشجویان علوم پزشکی تهران در سال تحصیلی 90-1389 انجام شد. روش بررسی: این مطالعه به روش مقطعی بر روی 720 نفر از دانشجویان دانشگاه علوم پزشکی تهران انجام گرفت. داده ها با استفاده از پرسشنامه جمع آوری گردید، تجزیه و تحلیل داده ها با استفاده از روشهای آماری توصیفی و آزمونLogistic regression و بابهره گیری از نرم افزار 16SPSS صورت پذیرفت. یافته ها: نتایج این مطالعه حاکی از آن بود که فراوانی مصرف سیگار و قلیان در دانشجویان به ترتیب 22 % ، 3/23% بوده است. همچنین پژوهش نشان داد که اصلی ترین علت مصرف سیگار و قلیان تفریح و سرگرمی بوده است. فراوانی مصرف سیگار و قلیان به ترتیب بطور معناداری در دانشکده های دندانپزشکی و داروسازی بیش از سایر دانشکده ها بود (001/0p<). بحث و نتیجه گیری: به طور کلی بر اساس یافته های مطالعه حاضر و مقایسه آن با یافته های سایر مطالعات صورت پذیرفته در یک دهه گذشته، میتوان گفت گرچه در چند سال اخیر شاهد روند رو به کاهش مصرف سیگار و قلیان بوده ایم، ولی بایستی همچنان برنامه های آموزش بهداشت در رابطه با پیشگیری ادامه پیدا کند. طراحی این برنامه ها از دوران مدرسه آغاز گردد و در دانشگاهها نیز تداوم یابد، همچنین پیشنهاد میشود در کنار اجرای این برنامه ها به مشکلات دانشجویان در زمینه تفریح و سرگرمی توجه گردد.

Title: سواد سلامت و بهره‌گیری از خدمات سلامت درجامعه شهری کرمان،1390

http://tbj.ssu.ac.ir/article-1-364-fa.html

سابقه و هدف: . با وجود افزایش نگرانی­ها درباره نتایج سواد سلامت پایین، تاثیرات سواد سلامت بر وضعیت سلامت و بهره­گیری از خدمات سلامت روشن نیست. هدف از این مطالعه ارزیابی سواد سلامت و تعیین ارتباط میان سواد سلامت و بهره­گیری از خدمات سلامت در جامعه شهری کرمان بود.   روش بررسی: در مطالعه ای که به روش مقطعی در شهر کرمان صورت گرفته است، 1000 نفر از افراد باسواد 17 سال و بالاتر مورد پرسشگری قرار گرفتند. به منظور جمع آوری اطلاعات از پرسشنامه استفاده گردید. برای آنالیز داده ها از شاخص ­ های توصیفی و نیز برای بررسی ارتباط متغیرهای از آزمون آماری کای دو، t و آنالیز واریانس یک طرفه و رگرسیون خطی استفاده گردید.   یافته ها: در حدود شصت درصد از افراد سطح سواد پایین(ناکافی و مرزی) داشتند. تحصیلات(001/0 p= ) و وضعیت بیمه (002/0 p= ) بیشترین ارتباط را با سواد سلامت نشان داد. سواد سلامت بر بهره­گیری از خدمات سلامت موثر بود. افرادی با نمره سواد سلامت بالا، به یک مرکز بهداشتی و درمانی مراجعه نموده بودند (006/0 p= ). بستری شدن در بیمارستان در افرادی با سواد سلامت پایین بیشتر گزارش شده بود(003/0 p= ). تاثیر سواد سلامت بر فاکتورهای بهره­گیری از خدمات سلامت بعد از حذف اثر عوامل مخدوش کننده، نیز تایید شد.   نتیجه گیری: سواد سلامت در کرمان پایین ارزیابی شد. بهبود سواد سلامت ممکن است استراتژی موثری در بهبود وضعیت سلامت و بهره­گیری از خدمات سلامت گردد. ارزیابی سواد سلامت برای بازتاب سطوح سواد سلامت افراد و تقاضای مناسب از نظام مراقبت سلامت مدرن باید در برنامه ها و سیاست گذاری های نظام سلامت لحاظ گردد. فرهنگ­سازی و آموزش برای بالا بردن سطح سواد سلامت جامعه و بهبود الگوی بهره­گیری از خدمات سلامت، همکاری و هماهنگی نزدیک نظام سلامت، آموزش و رسانه­ها را می­طلبد.      این مقاله حاصل پایان نامه جهت دریافت مدرک کارشناسی ارشد است که طرح پژوهشی آن از سوی معاونت پژوهشی دانشگاه علوم پزشکی کرمان تصویب شده است.

Title: تأثیر آموزش بهداشت یائسگی توسط داوطلبان سلامت بر دانش زنان اجتماع؛ یک مطالعه ی مداخله ای

http://tbj.ssu.ac.ir/article-1-365-fa.html

سابقه و هدف: مطالعات اندکی روی نقش داوطلبان سلامت در آموزش مراقبت از خود به زنان وجود دارد. از این رو، مطالعه ی حاضر به منظور ارزیابی نقش داوطلبان سلامت در آموزش بهداشت یائسگی به زنان اجرا شده است.   روش بررسی: در این مطالعه ی نیمه تجربی،48 داوطلب سلامت و 480 نفر از زنان تحت پوشش آنان در 8 مرکز از 70 مرکز بهداشت شهری در شیراز شرکت داشتند.ابتدا یک کارگاه آموزشی 8 ساعته طی دو روز برای داوطلبان سلامت اجرا شد. پس از آن از داوطلبان درخواست شد تا به آموزش بهداشت یائسگی به زنان بپردازند. پرسشنامه ای خود-ایفا برای جمع آوری داده های جمعیت شناختی استفاده شد. سطوح دانش داوطلبان و زنان قبل و بعد از مداخلات آموزشی با استفاده از یک آزمون کتبی (ضریب کودر-ریچاردسون 20 برابر با 66/0)، مشتمل بر 30 سؤال چندگزینه ای، اندازه گیری گردید. داده های پژوهش با استفاده از نسخه ی 5/11 نرم افزار SPSS و کاربرد آزمون های ناپارامتریک مجذور کای، ویلکاکسون، و من ویتنی پردازش و تحلیل شدند.   یافته ها: میانگین نمرات دانش داوطلبان سلامت در گروه آزمایش، از 1/17 قبل از کارگاه آموزشی به 2/29 بعد از کارگاه افزایش یافته بود ) 001/0 > P ) . میانگین های نمرات دانش داوطلبان در گروه شاهد در پیش آزمون و پس آزمون، به ترتیب 3/16 و 7/14 بودند. دانش زنان در گروه آزمایش به نحو بارزی از 4/12، قبل از آموزش توسط داوطلبان، به 4/25 پس از آن افزایش یافت (001/0 > P ) . سطوح دانش زنان در گروه شاهد در پیش آزمون و پس آزمون به ترتیب 1/15 و 9/14 بود.   نتیجه گیری: یافته ها نشان دهنده ی اثربخشی مداخله ی آموزشی در توانمندسازی داوطلبان، همچنین موفقیت داوطلبان در آموزش سلامت یائسگی به زنان در اجتماع آنان است. از این رو، پیشنهاد می شود متولیان سلامت با برنامه ریزی مناسب به توسعه ی این ظرفیت اجتماعی بپردازند و از آنان برای ارتقای سلامت زنان استفاده نمایند.
